# Supplementary material for: Principal Amalgamation Analysis for Microbiome Data
Source: Genes (Basel). 2022 Jun 24;13(7):1139. doi: 10.3390/genes13071139 (PMC9318429; doi:10.3390/genes13071139)
Supplement: Supplementary file 1 [file genes-13-01139-s001.zip › genes-1733484-supplementary.pdf]

Supplementary Material for "Principal Amalgamation Analysis for Microbiome Data"

Yan Li, Gen Li and Kun Chen*

June 13, 2022

*Corresponding author; kun.chen@uconn.edu.

A Visualization Example

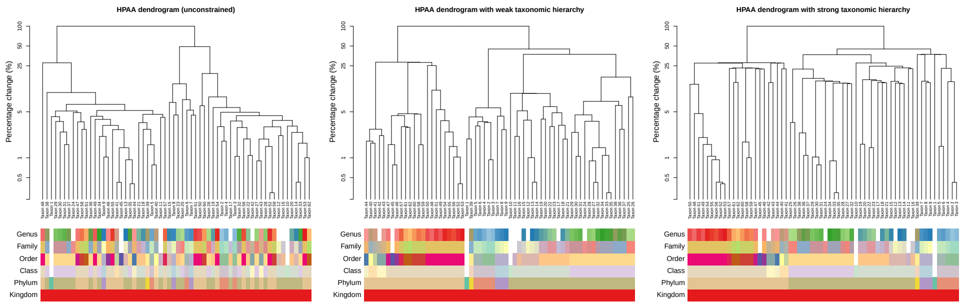

Figure S1: The NICU data: HPAA dendrograms with SDI and different constraints on taxonomic hierarchy.

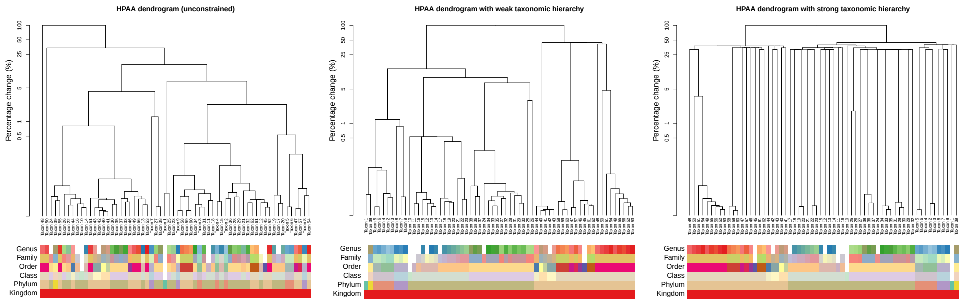

Figure S2: The NICU data: HPAA dendrograms with Bray-Curtis and different constraints on taxonomic hierarchy.

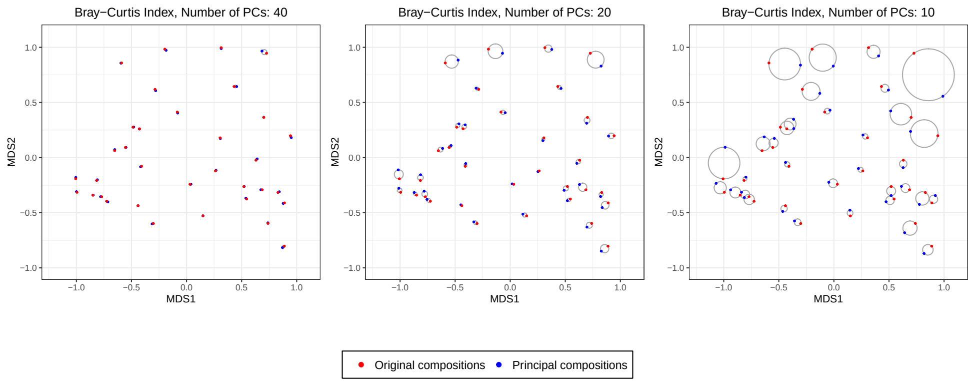

Figure S3: The NICU data: 2D NMDS ordination plots for comparing original data and different numbers of principal compositions from HPAA with Bray-Curtis and weak taxonomic hierarchy.

B Simulation

We first compare the computation efficiency of HPAA and amalgam, and ACLUST. To explore the effect of the dimensionality and the the sample size, we generate compositional data with $n = 100, p \in \{25, 50, 100, 200, 400, 800\}$ and $n \in \{25, 50, 100, 200, 400\}, p = 400$, respectively, using the amalgam package. The package uses Poisson distribution with $\lambda = 100$ to generate raw count at each matrix entry and then converts the counts into compositional data. For amalgam, the number of amalgamations is fixed at $k = 3$ as the computation time of amalgam greatly increases with the number of amalgamations, while we use the unconstrained HPAA methods with different loss functions to generate entire paths of amalgamations. We remark that the unconstrained HPAA is more time consuming than its constrained counterpart, as at each step the former always needs to solve the amalgamation problem over a larger active set. Each setting is repeated 10 times and the average running time of each method is reported in Figure S4. We have omitted ACLUST since it cannot even handle moderate dimension comparable to the real data example with $p = 60$. The results show that HPAA is computationally efficient and scales well with the increase of the dimension or the sample size. In contrast, amalgam and ACLUST are very computationally intensive even for moderately large p or n, making them unsuitable for large-scale microbiome studies.

We also compare different dimension reduction methods on how well they preserve the between-sample distance pattern, which is very importance in many biological applications. Here we simulate data to mimic the HIV infection dataset, presented in Section 4.2 of the main paper. Specifically, each raw count vector is generated from the multinomial distribution with the total count being 10,000 and the probabilities being the average proportions of the top $p = 20$ taxon in the HIV dataset; the count vector is then normalized to be compositional. The same taxonomic tree structure as in the HIV dataset is used. Three sample sizes are considered, i.e., $n \in \{50, 100, 200\}$. We use HPAA with weak taxonomic hierarchy, amalgam, and ACLUST to reduce the simulated data to $k = 10$ dimensions. The taxonomic guidance is one of the essential components in our proposed methods, without which the computational cost becomes much higher and the results of amalgamation are not interpretable.. The prevalence-based filtering method is also included as the baseline, which simply keeps the top 10 most prevalent taxa. In each simulation, the mean squared error (MSE) of the two between-sample distance matrices, computed from either the original data or the reduced data based on Bray-Curtis dissimilarity, is computed for each method. The procedure is repeated 100 times under each setting. The results are shown in Figure S5, in which the boxplots are constructed from the relative mean squared errors (RMSE) using the prevalence-based filtering method as the baseline, i.e., each RMSE is computed as the MSE divided by the median of the prevalence-based method (so that the boxplots of the prevalence-based method are with the median equal to 1). It is clear that all three HPAA methods with different loss functions outperform the baseline, amalgam, and ACLUST. PAA with the Bray-Curtis loss performs the best, as

it directly aims at preserving the Bray-Curtis dissimilarity. To our surprise, the `amalgam` method performs worse than the baseline, which may be due to its requirement of zero-replacement and log-ratio transformation and the slow convergence of its genetic algorithm. Moreover, ACLUST performs even worse than `amalgam`.

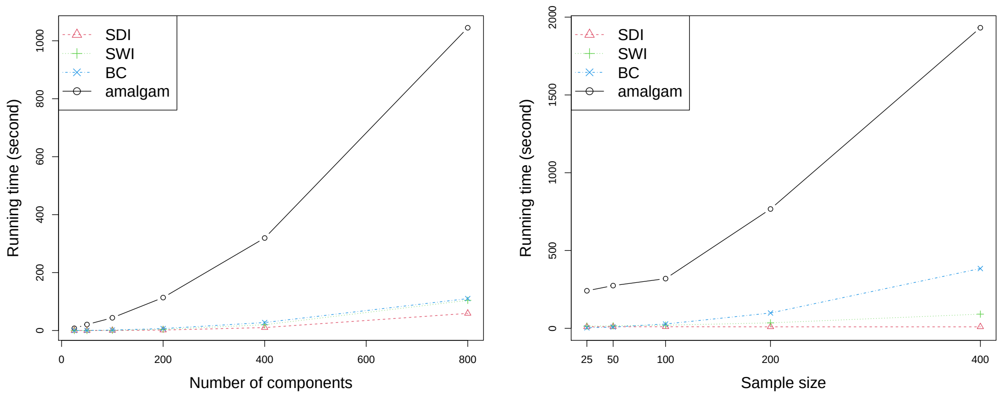

(a) Effect of dimension (b) Effect of sample size

Figure S4: Simulation: Average running time (in second) of HPAA methods with Simpson's index (SDI), Shannon's index (SWI) and Bray-Curtis dissimilarity (BC), and the `amalgam` method by Quinn and Erb (2020).

References

Greenacre, M. (2020). Amalgamations are valid in compositional data analysis, can be used in agglomerative clustering, and their logratios have an inverse transformation. Applied Computing and Geosciences 5, 100017.

Quinn, T. P. and I. Erb (2020). Amalgams: data-driven amalgamation for the dimensionality reduction of compositional data. NAR Genomics and Bioinformatics 2(4), lqaa076–lqaa076.

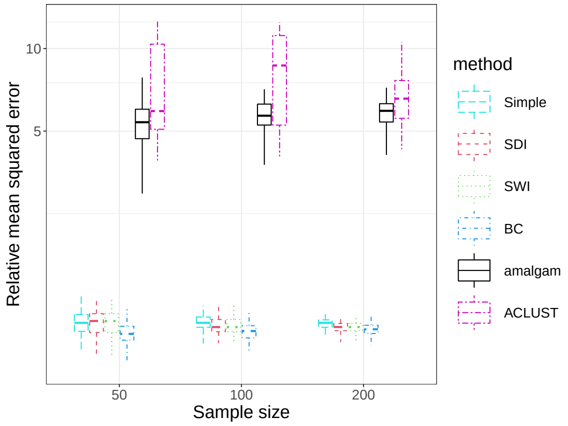

Figure S5: Simulation: Accuracy in preserving between-sample Bray-Curtis dissimilarity after dimension reduction. Five dimension reduction methods are considered: simple prevalence-based filtering method (Simple), HPAA methods with Simpson's index (SDI), Shannon's index (SWI) and Bray-Curtis dissimilarity (BC), the `amalgam` method by Quinn and Erb (2020), and ACLUST by Greenacre (2020). For each of compression, boxplots are constructed for the relative mean squared errors, i.e., mean squared error divided by the median of the Simple approach.
